# Lomax exponential distribution with an application to real-life data

**DOI:** 10.1371/journal.pone.0225827

**Published:** 2019-12-11

**Authors:** Muhammad Ijaz, Syed Muhammad Asim, Alamgir

**Affiliations:** Department of Statistics, University of Peshawar, Peshawar, KPK, Pakistan; Tongii University, CHINA

## Abstract

In this paper, a new modification of the Lomax distribution is considered named as Lomax exponential distribution (LE). The proposed distribution is quite flexible in modeling the lifetime data with both decreasing and increasing shapes (non-monotonic). We derive the explicit expressions for the incomplete moments, quantile function, the density function for the order statistics etc. The Renyi entropy for the proposed distribution is also obtained. Moreover, the paper discusses the estimates of the parameters by the usual maximum likelihood estimation method along with determining the information matrix. In addition, the potentiality of the proposed distribution is illustrated using two real data sets. To judge the performance of the model, the goodness of fit measures, AIC, CAIC, BIC, and HQIC are used. Form the results it is concluded that the proposed model performs better than the Lomax distribution, Weibull Lomax distribution, and exponential Lomax distribution.

## Introduction

In probability theory, it has been a usual practice for the last few years to modify the existing probability distributions so as to improve the flexibility of the existing models. These modifications are based on different methods such as increasing the number of parameters, making some transformation in the original distribution, proper mixing of two distributions etc. The main goal of such modifications is to improve the flexibility of the classical models. Motivating from the above methods, Ghitany and Al-Awadhi [1] proposed a compound form of the Lomax distribution with exponential distribution. Cordeiro et al. [2] modified the gamma-G family of distributions. Zografos et al. [3] employed the cumulative distribution function (Cdf) of the Lomax distribution as a baseline distribution. Lemonte et al. [4] and Lai et al. [5] used the idea of combining two distributions. Lemonte et.al [6] demonstrated the idea of Mcdonald-G family of distribution with a Lomax baseline function. Ibrahim et al. [7] modified the Lomax distribution by producing the real number to the power of the cumulative distribution function (Cdf) of Lomax distribution. Ashour and Eltehiwy [8], Merovci and Puka [9] and Khan et al. [10] utilized the well-known method that is the transmutation technique to generate new probability distributions.

In this paper, we propose a modification to the Lomax distribution. The Lomax distribution is defined as:

let a positive random variable Y has the Lomax distribution with parameters *a* and *b*, then the cumulative distribution function (Cdf) takes the form

$$F\left(y\right)=1-{\left[1+\left(\frac{y}{b}\right)\right]}^{-a},\;y>0\;and\;a,b>0$$ (1)

where *a* and *b* are the shape and scale parameters respectively. The probability density function related to (1) is given by

$$f\left(y\right)=\frac{a}{b}{\left[1+\left(\frac{y}{b}\right)\right]}^{-\left(a+1\right)},\;y>0;a,b>0$$ (2)

The above probability distribution has been modified by many researchers. For example, Cordeiro et al. [2] explored the gamma-Lomax distribution and discussed its applications to real data sets. Lemonte et al. [6] presented an extended Lomax distribution. Ibrahim et al. [7] produced a new three parameters probability distribution and referred to it as exponentiated Lomax distribution. Ashour and Eltehiwy [8] discussed the new modification to the Lomax distribution and termed it as a transmuted exponentiated Lomax distribution. Tahir et al. [11] discussed the Weibull Lomax distribution with applications to applied data.

The Lomax distribution is a heavily skewed probability distribution that plays a vital role in modeling the lifetime data sets produced in business, computer science, medical and biological sciences, engineering, economics, income and wealth inequality, Internet traffic and reliability modeling. The Lomax or Pareto II distribution have been applied to model the data related to income and wealth [12, 13], the distribution of computer files on server [14], reliability and life testing [15] etc. The Lomax distribution is an alternative to the exponential distribution when the data are heavily tailed [16]. The Lomax distribution has also been applied to record values by Ahsanullah [17]. El-Bassiouny et al. [18] investigated the exponential Lomax distribution. Afify et al. [19] defined the transmuted Weibull-Lomax distribution with real-world applications. For other probability distributions and their applications to different fields, we refer to see [20–31], and [32–36] respectively.

In reliability theory where one deals with life testing experiments, most of the data sets result in non-monotonic hazard rate shapes. In such situations, the existing distributions fail to provide an adequate fit to the data. The main goal of this paper is to provide a new probability model that would be more flexible which adequately represents the data sets and have tractable statistical properties. The proposed model shall refer to as Lomax exponential distribution. The proposed model is produced using the transformation

$$X=Ye^{Y}$$

in the Lomax distribution. In the following section we have derived different statistical properties including hazard rate function, survival function, quantile function, moments, order statistics, parameter estimation, Renyi entropy, and asymptotic confidence bounds of the proposed model. We have further explored applications of the proposed model with two real data sets in addition to a simulation study.

## Lomax exponential (LE) distribution

Let a random variable Y has the Lomax exponential distribution with parameters *a* and *b*. The parameters *a* and *b* are the shape and scale parameters respectively. The cumulative distribution function of the Lomax exponential distribution is given by

$$F\left(y\right)=1-{\left[1+\left(\frac{yexp\left(y\right)}{b}\right)\right]}^{-a},\;y>0$$ (3)

The corresponding probability function to (3) is given as

$$f\left(y\right)=\frac{a}{b}\left(y+1\right)exp\left(y\right){\left[1+\left(\frac{yexp\left(y\right)}{b}\right)\right]}^{-\left(a+1\right)},\;y>0$$ (4)

The hazard rate function and survival functions respectively are defined as

$$h\left(y\right)=\frac{aexp\left(y\right)}{b\left[1+\left(\frac{yexp\left(y\right)}{b}\right)\right]}$$ (5)

$$S\left(y\right)={\left[1+\left(\frac{yexp\left(y\right)}{b}\right)\right]}^{-a}$$ (6)

**Fig 1:**
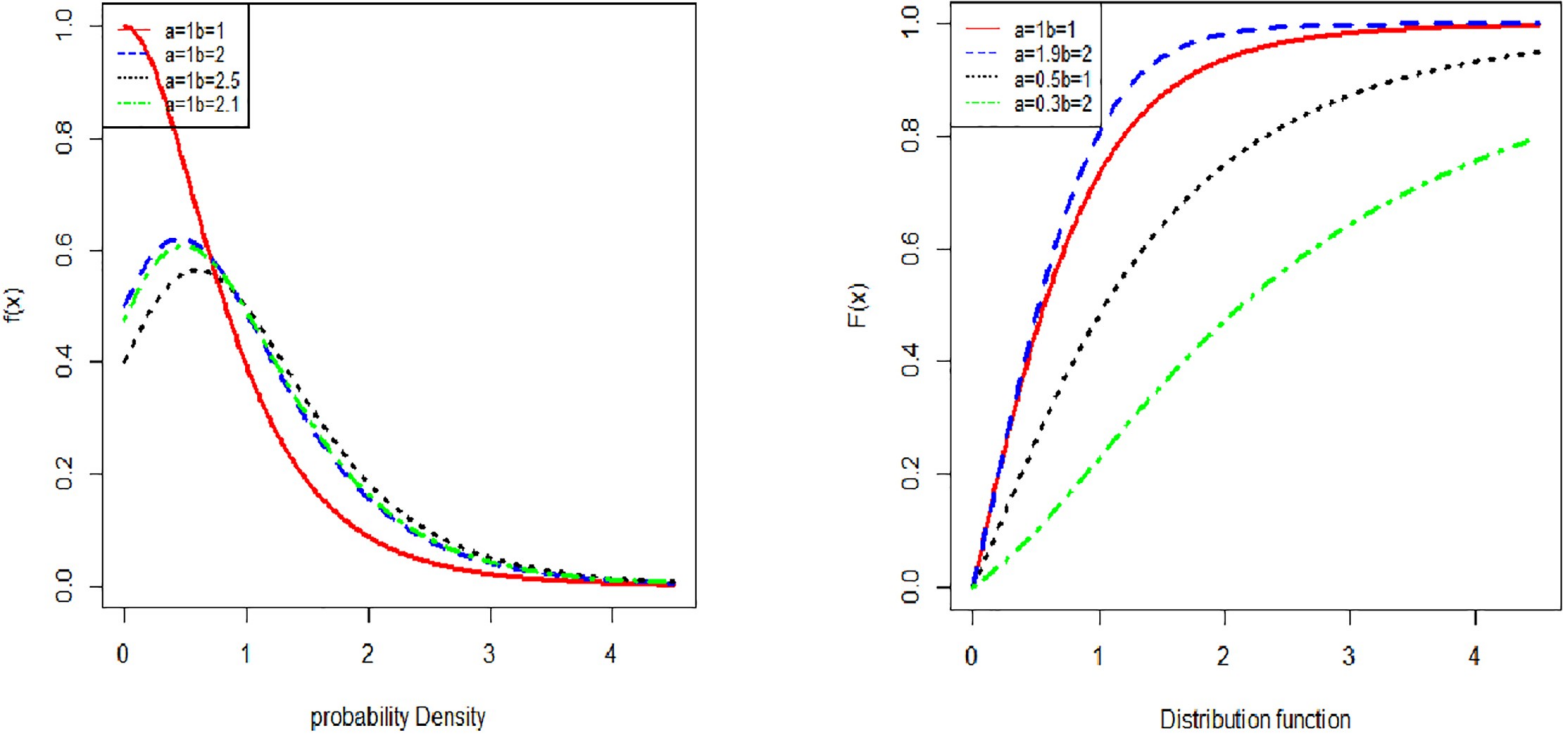
Shapes of the Pdf and Cdf of Lomax exponential distribution.

Fig 1 shows the graphical representation of the probability density function and cumulative distribution function, with different parameter values.

### The behavior of the hazard rate function

**Theorem 1.** The behavior of the hazard rate function of Lomax exponential (*a*,*b*) distribution *h*(*y*) is studied by taking the derivative of the hazard rate function in Eq (5) and is given by

$$h^{\prime }(y)=\frac{d}{dy}\left[\frac{\left(y+1)a\exp(y\right)}{b\left[1+\left(\frac{y\exp(y)}{b}\right)\right]}\right]$$

Simplifying we get

$$h^{\prime }(y)=-\frac{ae^{y}\left(e^{y}-by-2b\right)}{{\left(ye^{y}+b\right)}^{2}}=\frac{ae^{y}\left(b(y+2)-e^{y}\right)}{{\left(ye^{y}+b\right)}^{2}}$$ (7)

The mode of the above expression is the roots of *h*′(*x*) = 0. If *b*>1, then *h*′(*x*) = 0 implies that the *h*(*x*) has a maximum at

$$y_{m}=-W\left(-\frac{1}{b\exp{\left(y\right)}^{2}}\right)-2,\;b\neq 0$$ (8)

where *W*(*z*) is the Lambert w function. The function *h*(*x*) is increasing if *h*′(*y*)>0 for *y*<*y*_*m*_ and *h*′(*y*)<0 for all values of *y*>*y*_*m*_. *h*(*x*) is decreasing if *h*′(*y*)<0 for *y*<*y*_*m*_ and *h*′(*y*)>0 for all values of *y*>*y*_*m*_.

**Fig 2:**
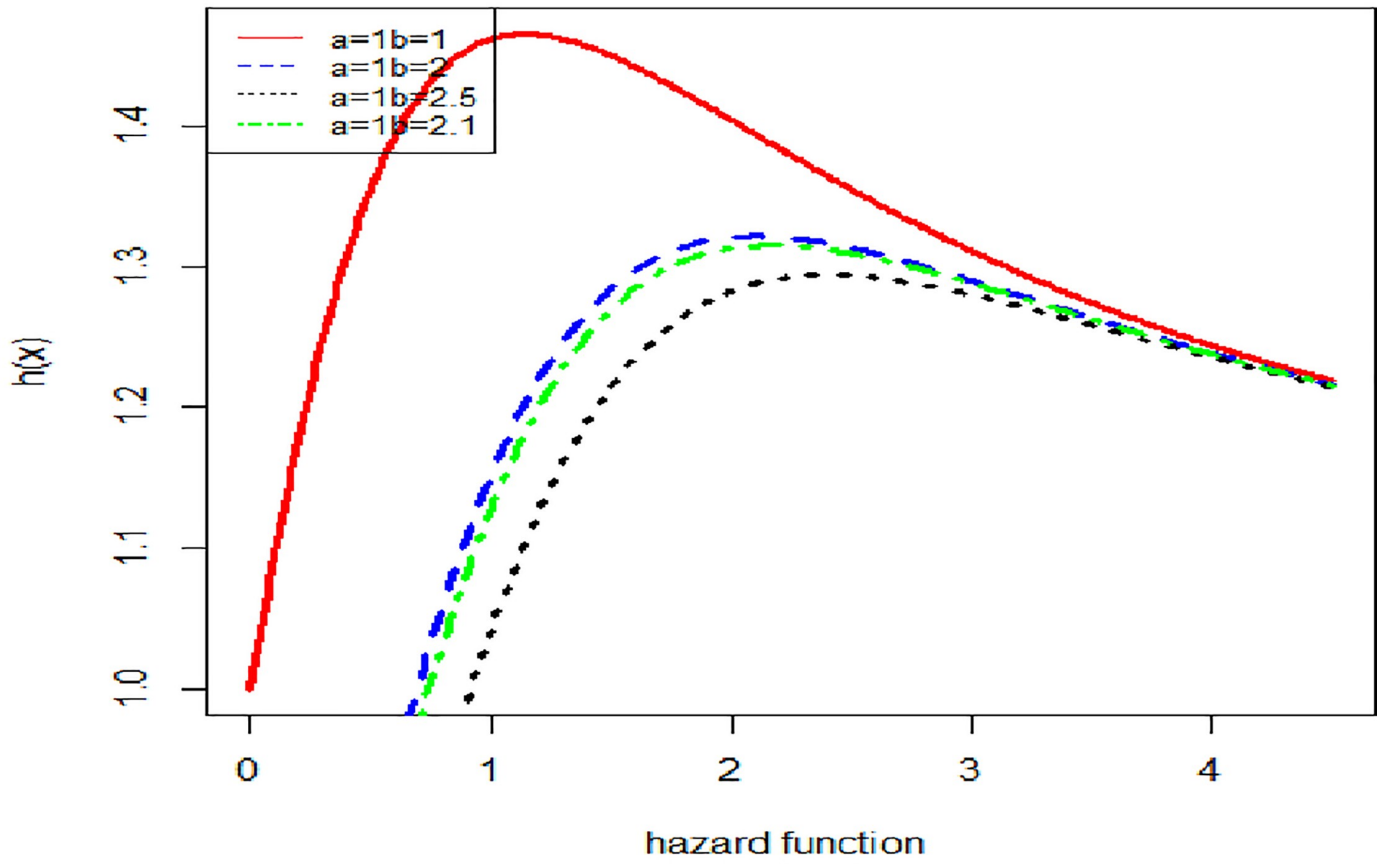
Shapes of the hazard rate function with different values of *b* when *a* = 1.

Fig 2 illustrates that the Lomax exponential distribution can model both monotonically and non-monotonically hazard rate shapes with different values of the parameter.

## Quantile function and median

The quantile function *Q*_(*FL*)_(*y*) of the *LE*(*a*,*b*) is the real solution of the following equation

$$1-{\left[1+\left(\frac{yexp(y)}{b}\right)\right]}^{-a}=u$$ (9)

where *u*~Uniform (0,1). Solving (9) for *y*, we have

$$y=W\left(\frac{b}{{\left(1-u\right)}^{1/a}}-b\right),$$ (10)

where W (.) is the product log function.

For calculating the median we have to put *u* = 0.5 in Eq (10) to have

$$y=W\left(\frac{b}{{\left(1-0.5\right)}^{1/a}}-b\right)$$ (11)

## Rth moment

**Theorem 2**. If *Y* has a Lomax exponential distribution with parameters *a and b* then the r^th^ moments (about the origin) of *X*, say $u_{r}^{\prime }$, does not exist.

$$\begin{array}{c} u_{r}=E(y^{r})=\int_{0}^{\infty }y^{r}f(y)dx,\;r=1,2,3\ldots \\ =\int_{0}^{\infty }y^{r}\frac{a}{b}\left(y+1\right)exp\left(y\right){\left[1+\left(\frac{yexp\left(y\right)}{b}\right)\right]}^{-\left(a+1\right)}dy. \end{array}$$

Using ${\left[1+\left(\frac{yexp\left(y\right)}{b}\right)\right]}^{-\left(a+1\right)}=\sum_{n=k=0}^{\infty }\frac{n^{k}}{b^{n}}\left(\begin{array}{c} -a-1\\ n \end{array}\right)y^{n+k}$ in the above expression to have

$$u_{r}^{\prime }=\sum_{n=k=0}^{\infty }\left(\begin{array}{c} -a-1\\ n \end{array}\right)\frac{n^{k}}{b^{n}}\int_{0}^{\infty }y^{r+n+k}\left(y+1\right)exp\left(y\right)dy$$ (12)

By solving (12) the integral in (12), we get expression for $u_{r}^{\prime }$ as follows

$$u_{r}=\sum_{n=k=0}^{\infty }\left(\begin{array}{c} -a-1\\ n \end{array}\right)\frac{n^{k}}{{\left(-1\right)}^{r+k+n}b^{n}}\left(\Gamma \left(r+k+n+2\right)+\Gamma \left(r+n+k\right)\right)$$ (13)

Hence the skewness and kurtosis can be defined by using the relation,

Skewness=E(y3)−3E(y)E(y2)+2E(y2)var32(y) (14)

$$kurtosis=\frac{E(y^{4})-4E(y)E(y^{3})+6E(y^{2})E^{2}(y)+3E^{4}(y)}{{\mathrm{var}}^{2}\left(y\right)}$$ (15)

where, var(*y*) = *E*(*y*^2^)−*E*^2^(*y*).

## Order statistics

Let *Y*_1_,*Y*_2_,…,*Y*_*n*_ be ordered random variables, then the probability density function (Pdf) of the *i*^*th*^ order statistics is given by,

$$f_{\left(i;n)(y\right)}=\frac{n!}{(i-1)!(n-i)!}f(y)F{\left(y\right)}^{\left(i-1\right)}{\left[1-F(y)\right]}^{\left(n-i\right)},$$ (16)

The 1^st^ and n^th^ order probability density function (pdf) of the *LE* can be obtained using (3) and (4) in (16) to have

$$f_{\left(1:n\right)}(x)=n{\left(\frac{a}{b}\left(y+1\right)exp\left(y\right){\left[1+\left(\frac{yexp\left(y\right)}{b}\right)\right]}^{-\left(a+1\right)}\right)}^{\left(n-1\right)}{\left[1+\left(\frac{yexp\left(y\right)}{b}\right)\right]}^{-a}$$ (17)

$$f_{\left(n:n\right)}(x)=n\left(\frac{a}{b}\left(y+1\right)exp\left(y\right){\left[1+\left(\frac{yexp\left(y\right)}{b}\right)\right]}^{-\left(a+1\right)}\right){\left[1-1+\left(\frac{yexp\left(y\right)}{b}\right)\right]}^{-a\left(n-1\right)}$$ (18)

## Parameter estimation

In this section, the usual method, that, the maximum likelihood estimation is used to find out the estimates of the unknown parameters of *LE*(*a*,*b*) based on complete information. Let us assume that we have a sample *Y*_1_,*Y*_2_,…,*Y*_*n*_ from *LE*(*a*,*b*). The Likelihood function is given by

$$L=\prod_{i=1}^{n}f\left(y_{i};a,b\right)$$ (19)

Substituting (4) in (19), we get

$$L=\prod_{i=1}^{n}\left(\frac{a}{b}\left(y+1\right)exp\left(y\right){\left[1+\left(\frac{yexp\left(y\right)}{b}\right)\right]}^{-\left(a+1\right)}\right)$$ (20)

By applying the natural logarithm to (20), the log-likelihood function is

$$l\left(y;a,b\right)=nlog\left(\frac{a}{b}\right)+\sum log\left(y_{i}+1\right)+\sum y_{i}-\left(a+1\right)\sum log\left(1+\frac{y_{i}\exp\left(y_{i}\right)}{b}\right)$$ (21)

Now computing the first partial derivatives of (21) and setting the results equal zeros, we have

$$\frac{d}{da}l\left(y;a,b\right)=\frac{n}{a}-\sum log\left(1+\frac{y_{i}\exp\left(y_{i}\right)}{b}\right)$$ (22)

$$\frac{d}{db}l\left(y;a,b\right)=-\frac{n}{ab}+\frac{\left(a+1\right)}{b}\sum \frac{y_{i}\exp\left(y_{i}\right)}{y_{i}\exp\left(y_{i}\right)+b}$$ (23)

$$\frac{d}{db^{2}}l\left(y;a,b\right)=\frac{n}{ab^{2}}+\sum \frac{{\left(y_{i}\exp\left(y_{i}\right)\right)}^{2}}{\left(1+\frac{y_{i}\exp\left(y_{i}\right)}{b}\right)b^{2}}$$ (24)

$$\frac{d}{dab}l\left(y;a,b\right)=-\frac{1}{b}\sum \left(\frac{y_{i}\exp\left(y_{i}\right)}{b+y_{i}\exp\left(y_{i}\right)}\right)$$ (25)

The above Eqs from (22) to (25) are not in closed form. For the solution of these explicit equations, we refer to using some iterative procedure such as Newton Raphson, Bisection methods, or some other to get the approximate maximum likelihood estimates (MLE) of these parameters.

## Asymptotic confidence bounds

Since the MLE of the unknown parameters *a*,*b* are not in closed forms, therefore, it is not possible to derive the exact distribution of the MLE. We have derived the asymptotic confidence bounds for the unknown parameters of *LE*(*a*,*b*) based on the asymptotic distribution of the MLE. For the information matrix, we find the second time partial derivatives of the Eqs from (22) to (25) and are given as

$$\frac{d}{da^{2}}l\left(y;a,b\right)=I_{11}=-\frac{n}{a^{2}}$$ (26)

$$\frac{d}{db^{2}}l\left(y;a,b\right)=I_{22}=\frac{n}{ab^{2}}+\sum \frac{{\left(y_{i}\exp\left(y_{i}\right)\right)}^{2}}{\left(1+\frac{y_{i}\exp\left(y_{i}\right)}{b}\right)b^{2}}$$ (27)

$$\frac{d}{dab}l\left(y;a,b\right)=I_{12}=-\frac{1}{b}\sum \left(\frac{y_{i}\exp\left(y_{i}\right)}{b+y_{i}\exp\left(y_{i}\right)}\right)$$ (28)

So that the observed information matrix is given by

$$I=-\left(\begin{array}{cc} I_{11} & I_{12}\\ I_{21} & I_{22} \end{array}\right)$$

Hence the variance-covariance matrix is approximated as

$$V=\left(\begin{array}{cc} v_{11} & v_{12}\\ v_{21} & v_{22} \end{array}\right)={\left(\begin{array}{cc} I_{11} & I_{12}\\ I_{21} & I_{22} \end{array}\right)}^{-1}$$

To obtain the estimate of V, we replace the parameters by the corresponding MLE^’^s to get

$$\overset{\wedge }{v}={\left(\begin{array}{cc} \overset{\wedge }{I_{11}} & \overset{\wedge }{I_{12}}\\ \overset{\wedge }{I_{21}} & \overset{\wedge }{I_{22}} \end{array}\right)}^{-1}$$ (29)

Using the above variance-covariance matrix, one can derive the (1 - β) 100% confidence intervals for the parameters *a* and *b* as following

$$\hat{a}\pm Z_{\frac{\beta }{2}}\sqrt{\mathrm{var}\left(\hat{a}\right)},\;\hat{b}\pm Z_{\frac{\beta }{2}}\sqrt{\mathrm{var}\left(\hat{b}\right)}.$$

where $Z_{\frac{\beta }{2}}$ is the upper ${\left(\frac{\beta }{2}\right)}^{th}$ percentile of the standard normal distribution.

## Renyi entropy

**Theorem 3.** If a random variable *X* has a *LE*(*a*,*b*), then the Renyi entropy *R*_*H*_(*x*) is defined by

$$\begin{array}{c} R_{H}(y)=\frac{1}{1-p}log\int_{0}^{\infty }f{\left(y\right)}^{p}dy\\ =\frac{1}{1-p}log\int_{0}^{\infty }{\left(\frac{a}{b}\left(y+1\right)exp\left(y\right){\left[1+\left(\frac{yexp\left(y\right)}{b}\right)\right]}^{-\left(a+1\right)}\right)}^{p}dy \end{array}$$ (30)

where

$$\begin{array}{r} {\left[1+\left(\frac{yexp\left(y\right)}{b}\right)\right]}^{-p\left(a+1\right)}=\sum_{n=0}^{\infty }\left(\begin{array}{c} -p\left(a+1\right)\\ n \end{array}\right){\left(\frac{yexp\left(y\right)}{b}\right)}^{n}\\ =\sum_{n=0}^{\infty }\frac{n^{k}}{b^{n}k!}\left(\begin{array}{c} -p\left(a+1\right)\\ n \end{array}\right)y^{n+k} \end{array}$$

By employing the result of the above expression in (30) we have

$$=\sum_{n=l=0}^{\infty }{\left(\frac{a}{b}\right)}^{p}\frac{n^{k}}{b^{n}k!}\left(\begin{array}{c} -p\left(a+1\right)\\ n \end{array}\right)\left(\begin{array}{c} l\\ y \end{array}\right)\int_{0}^{\infty }y^{l+n+k}exp\left(py\right)dy$$ (31)

Solving the function under the integral sign, finally we get

$$R_{H}(y)=\sum_{n=0}^{\infty }\sum_{l=0}^{\infty }\left(\begin{array}{c} p\\ l \end{array}\right){\left(\frac{a}{b}\right)}^{p}\frac{n^{k}}{b^{n}k!}\left(\begin{array}{c} -p(a+1)\\ n \end{array}\right)\frac{1}{1-p}\log\left[\frac{\Gamma (l+n+k+1)}{{\left(-p\right)}^{k+n+k}}\right]$$ (32)

## Applications

In this section, we provide an application of the LE distribution to two real data sets to illustrate its usefulness and compare its goodness-of-fit with other invariant forms of the Lomax distribution including the Weibull Lomax (WL) [11], Exponential Lomax (EL) [18], and the Lomax (L) [37], by using Kolmogorov–Smirnov (K–S) statistic, Akaike Information Criterion (AIC), Consistent Akaike Information Criterion (CAIC), Bayesian Information Criterion (BIC), and Hannan Quinn information Criterion (HQIC). Formulae of these criteria are given by

$$AIC=-2L(\hat{\psi };y_{i})+2p,\;AICc=AIC+\frac{2p(p+1)}{n-p-1},\;CAIC=-2L+P\left\{\log(n)+1\right\},$$

$$BIC=P\log\left(n\right)-2L(\hat{\psi };y_{i}),\;HQIC=-2L_{max}+2P\log\left\{\log(n)\right\}.$$

where *L* is the maximized likelihood function and *y*_*i*_ is the given random sample, $\hat{\psi }$ is the maximum likelihood estimator and *p* is the number of parameters in the model.

### Data set 1: Losses due to wind catastrophes

The first data set represents the losses due to wind catastrophes recorded in 1977 used by Hogg and Klugman [38]. The data set consists of 40 observations that were recorded to the nearest $1,000,000 and include only losses of $2,000,000 or more. The data set values are as follows (in millions of dollars):

2,2,2,2,2,2,2,2,2,2,2,2,3,3,3,3,4,4,4,5,5,5,5,6,6,6,6,8,8,9,15,17,22,23,24,25,27,32,43.

### Data set 2: Breaking stress of carbon fibers

The second real data set represent the failure times of 84 aircraft windshield. This data is taken from an article published by [18]. The data points are as follows: 3.70,2.74,2.73,2.50,3.60,3.11,3.27,2.87,1.47,3.11,4.42,2.41,3.19,3.22,1.69,3.28,3.09,1.87,3.15,4.90,3.75,2.43,2.95,2.97,3.39,2.96,2.53,2.67,2.93,3.22,3.39,2.81,4.20,3.33,2.55,3.31,3.31,2.85,2.56,3.56,3.15,2.35,2.55,2.59,2.38,2.81,2.77,2.17,2.83,1.92,1.41,3.68,2.97,1.36,0.98,2.76,4.91,3.68,1.84,1.59,3.19,1.57,0.81,5.56,1.73,1.59,2.00,1.22,1.12,1.71,2.17,1.17,5.08,2.48,1.18,3.51,2.17,1.69,1.25,4.38,1.84,0.39,3.68,2.48,0.85,1.61,2.79,4.70,2.03,1.80,1.57,1.08,2.03,1.61,2.12,1.89,2.88,2.82,2.05,3.65.

Table 1 represent the maximum likelihood estimates and Table 2 represent the goodness of fit measures AIC, CAIC, BIC, and HQIC of the Lomax exponential distribution for the wind catastrophes data. Table 3 represent the maximum likelihood estimates and Table 4 represent the goodness of fit measures AIC, CAIC, BIC, and HQIC using breaking stress of carbon fibers data. In general, the model is to be considered the best one among others for which these (AIC, CAIC, BIC, and HQIC) statistics values are small. From Table 2 and Table 4, it is evident that the LE model leads to the preferable fit over the Lomax, Weibull Lomax, and Exponential Lomax distribution.

**Table 1:** Maximum likelihood estimates for data set 1.

Model	Estimates			
*LE*(*a*,*b*)	0.1104961	5.6340882	_	_
*WL*(*a*,*b*,*c*,*d*)	2.8345778	1.9742578	1.0284592	0.2073842
*L*(*a*,*b*)	2.259102	13.107217	_	_
*EL*(*a*,*b*,*c*)	0.975232421	0.062429585	0.008794612	_

**Table 2:** Goodness of fit Criteria: AIC, CAIC, BIC, HQIC for Data set 1.

Model	*AIC*	*CAIC*	*BIC*	*HQIC*
*LE*(*a*,*b*)	243.7959	244.1293	247.1231	244.9897
*WL*(*a*,*b*,*c*,*d*)	249.5339	250.7104	256.1881	251.9214
*L*(*a*,*b*)	252.6833	253.0166	256.0104	253.877
*EL*(*a*,*b*,*c*)	255.0222	255.7079	260.0129	256.8128

**Table 3:** Maximum likelihood estimates for data set 2.

Model	Estimates			
*LE*(*a*,*b*)	1.125319	34.175778	_	_
*WL*(*a*,*b*,*c*,*d*)	0.01968493	1.39764915	1.79715292	2.91573568
*L*(*a*,*b*)	9.44236	23.14359	_	_
*EL*(*a*,*b*,*c*)	4.1176675	1.5270909	0.0114609	_

**Table 4:** Goodness of fit Criteria: AIC, CAIC, BIC, HQIC for data set 2.

Model	*AIC*	*CAIC*	*BIC*	*HQIC*
*LE*(*a*,*b*)	263.2525	263.3988	268.1378	265.2175
*WL*(*a*,*b*,*c*,*d*)	265.3037	265.8037	275.0743	269.2338
*L*(*a*,*b*)	341.1852	341.3315	346.0705	343.1502
*EL*(*a*,*b*,*c*)	264.2428	264.5391	271.5708	267.1903

**Fig 3:**
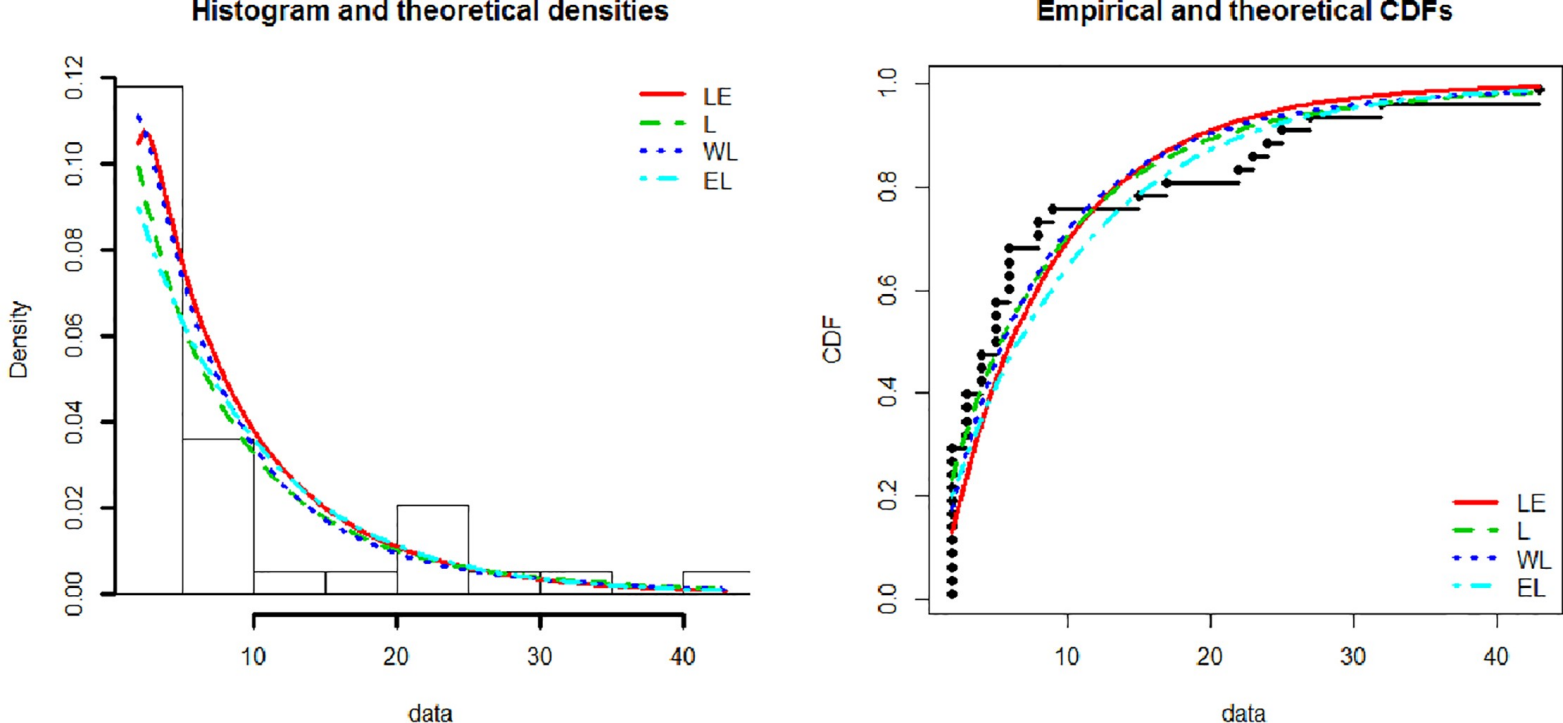
Theoretical and empirical Pdf and Cdf of LE for data set 1.

**Fig 4:**
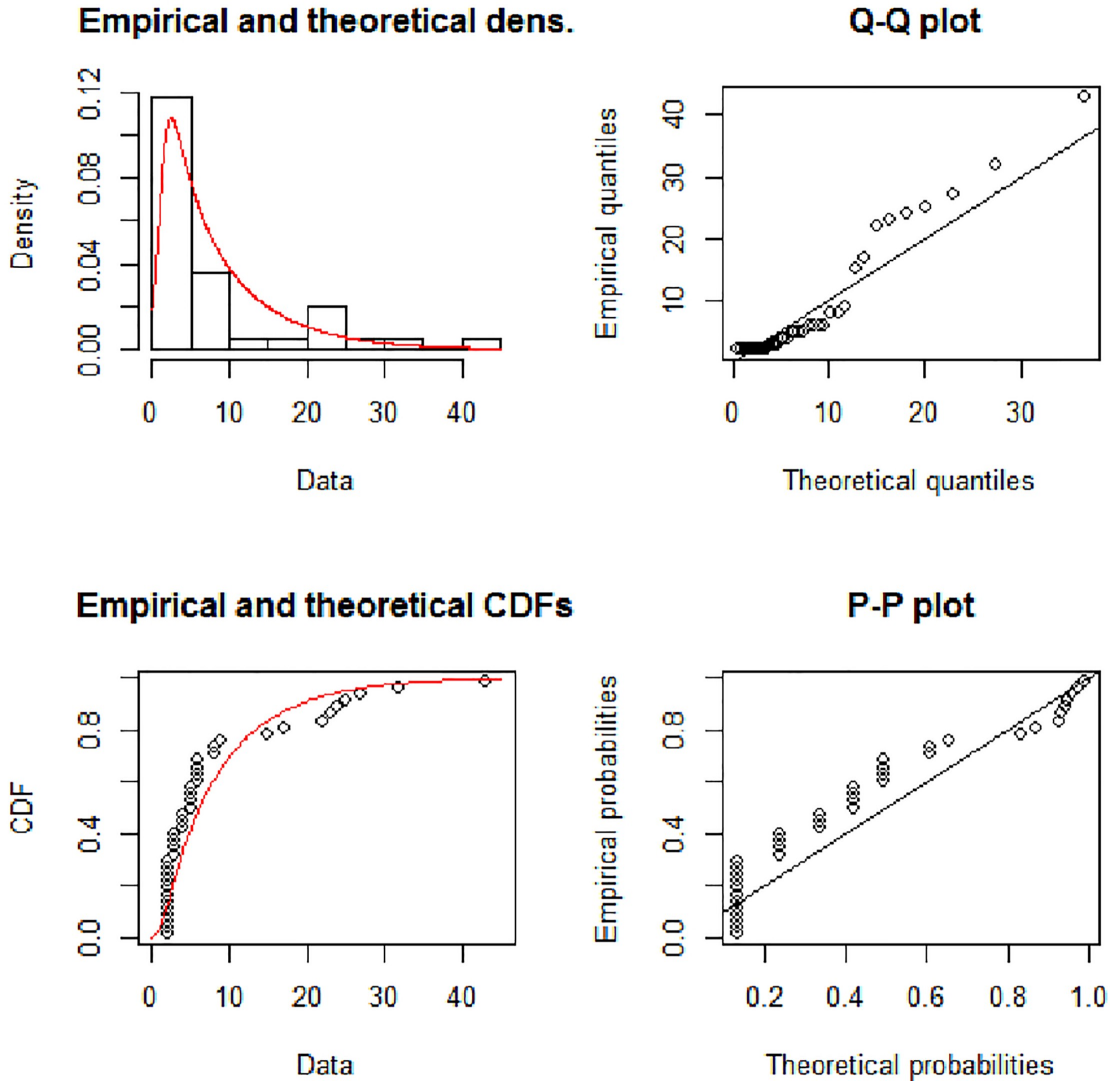
Theoretical and empirical Pdf and Cdf with Q-Q plot and P-P plot for LE for data set 1.

**Fig 5:**
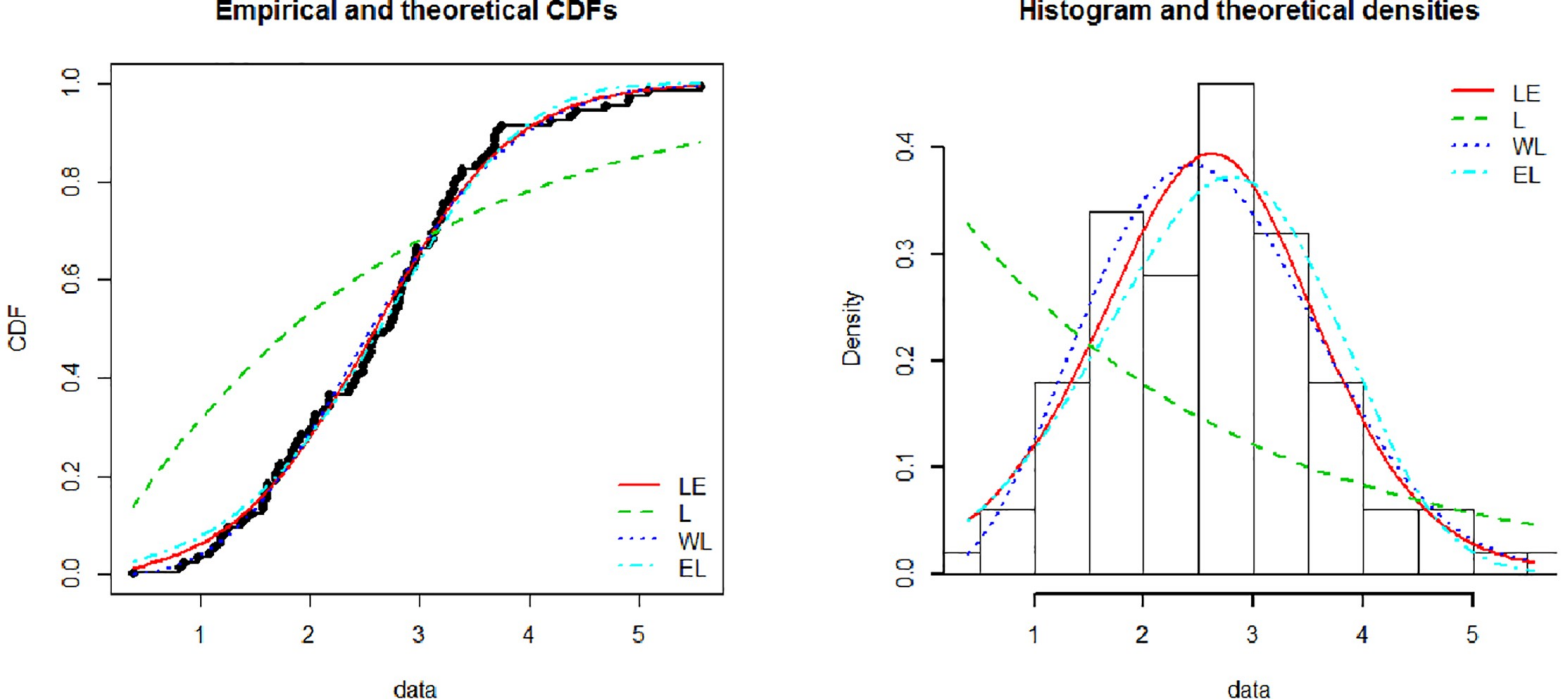
Theoretical and empirical Pdf and Cdf of LE for data set 2.

**Fig 6:**
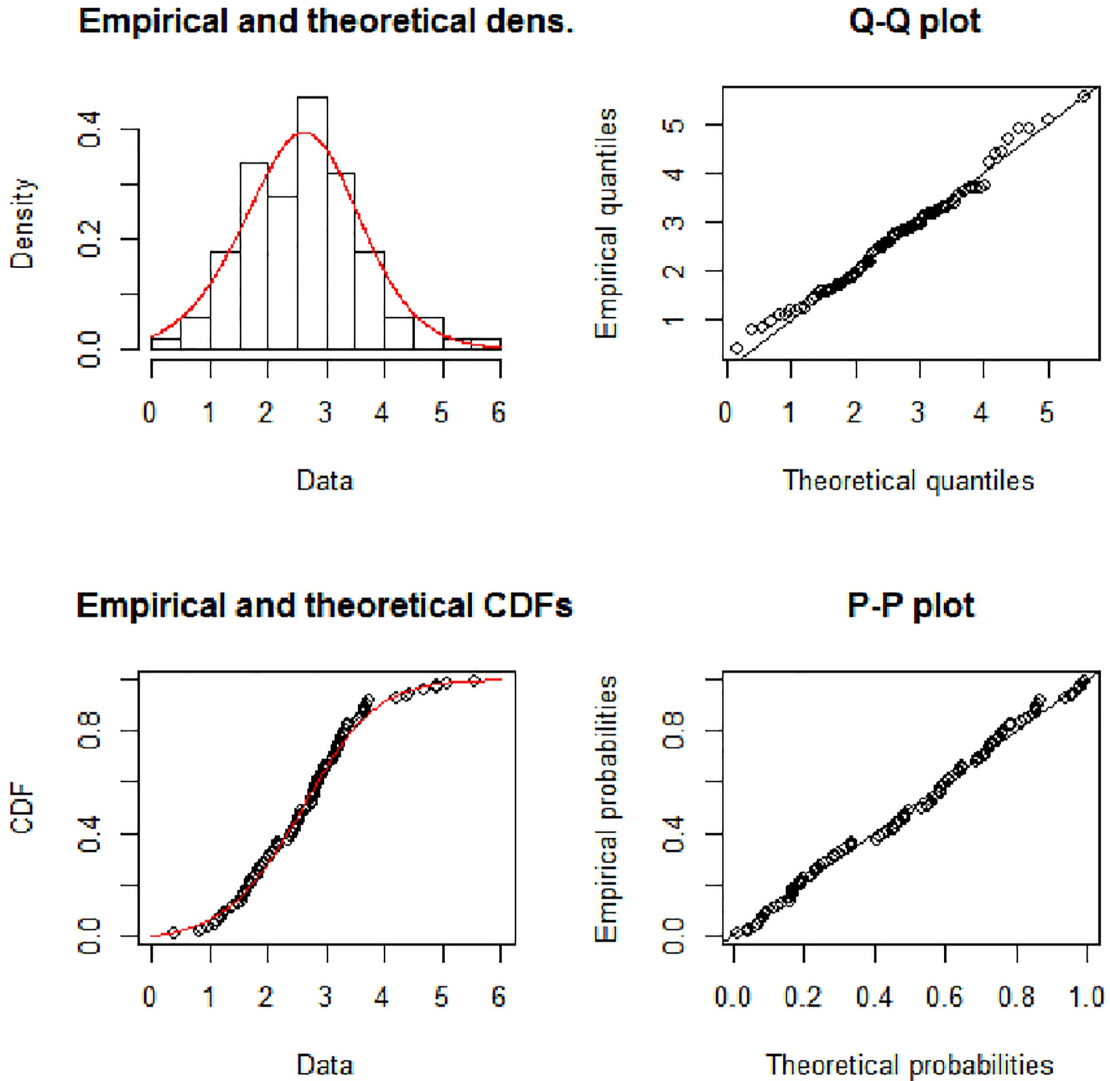
Theoretical and empirical Pdf and Cdf with Q-Q plot and P-P plot for LE for data set 2.

Fig 3 show the theoretical and empirical probability density function (Pdf) and cumulative distribution function (Cdf) and Fig 4 provides the Q-Q plot and P-P plot of the Lomax exponential for data set 1. Fig 5 shows the theoretical and empirical probability density function (Pdf) and cumulative distribution function (Cdf) and Fig 6 provides the Q-Q plot and P-P plot of the Lomax exponential for data set 2. It is evident that the LE distribution fitted the line very well as compared to others

### Simulations

Expression (11) can be easily used to draw random data from *LE*(*a*,*b*) distribution. The experiment is repeated for 100 times with a sample of size *n* = 30, 60, and 90 for different values of the parameter. The average bias and Mean square error (MSE) are given in Table 5. The results reveal that increase in the sample size results in a decrease in both the bias and MSE. The mathematical form of the mean square error and bias are as follows:

$$MSE=\frac{1}{W}{\sum_{i=1}^{W}\left(\hat{\alpha_{i}}-\alpha \right)}^{2}$$

$$Bias=\frac{1}{W}\sum_{i=1}^{W}\left(\hat{\alpha_{i}}-\alpha \right)$$

**Table 5:** Mean bias and MSE of *LE*(*a*,*b*) distribution.

*a*	*b*	*n*	*Mse*(*a*)	*Mse*(*b*)	*bias*(*a*)	*bias*(*b*)
2	0.1	30	43.37569	0.2849909	3.519714	0.2674605
		60	2.797546	0.01698649	0.8652669	0.0698271
		90	2.547909	0.0147992	0.7191287	0.05946671
2	1.5	30	7.948621	7.12233	0.8635859	0.8316998
		60	1.906238	1.895496	0.2121933	0.328816
		90	0.06762601	0.1207555	0.02700033	0.2979983
2	2	30	16.30946	31.8214	1.998589	2.647095
		60	6.757092	10.25353	1.384559	1.652988
		90	0.8164857	1.4583	0.1488166	0.332031
0.01	0.21	30	1.136051e-05	3.127487	0.002299238	1.507933
		60	8.759477e-07	0.5738629	0.0009260684	0.7550524
		90	2.464595e-07	0.01949125	0.0004921424	0.1385804
0.01	0.30	30	1.223512e-05	4.177988	0.003459386	2.036716
		60	4.526665e-07	0.2298188	0.0006659029	0.4761458
		90	2.010531e-07	0.03170673	0.0004390346	0.1755684
0.01	0.29	30	1.285701e-06	3.607665	0.0007714635	1.78106
		60	3.925089e-07	0.08345893	0.0004569719	0.2779985
		90	2.014616e-07	0.02836001	0.0004394908	0.1657713

### Total time on the test (TTT)

The TTT plot plays an important role in identifying the appropriate model to fit the given data in respect of the failure rates. This plot tells us the different forms of the failure rate. If the TTT plot has a straight line (diagonal), this indicates that the given data has a constant failure rate. The failure rates will be increase if this plot is concave and decreases if it is convex. For the bath-tub shape, this plot first decreases and then increases. Similarly, if the failure rates follow some inverted bath-tub shape, then it will be first concave and then convex. The TTT plot is determined by using the following formula

$$G\left(\frac{r}{n}\right)=\frac{\sum_{i=1}^{r}x_{i:n}+\left(n-r\right)x_{i:n}}{\sum_{i=1}^{n}x_{i:n}},\;r=x_{i:n}=1,2,3,\ldots n$$

where *x*_*i*:*n*_ are the order statistics.

**Fig 7:**
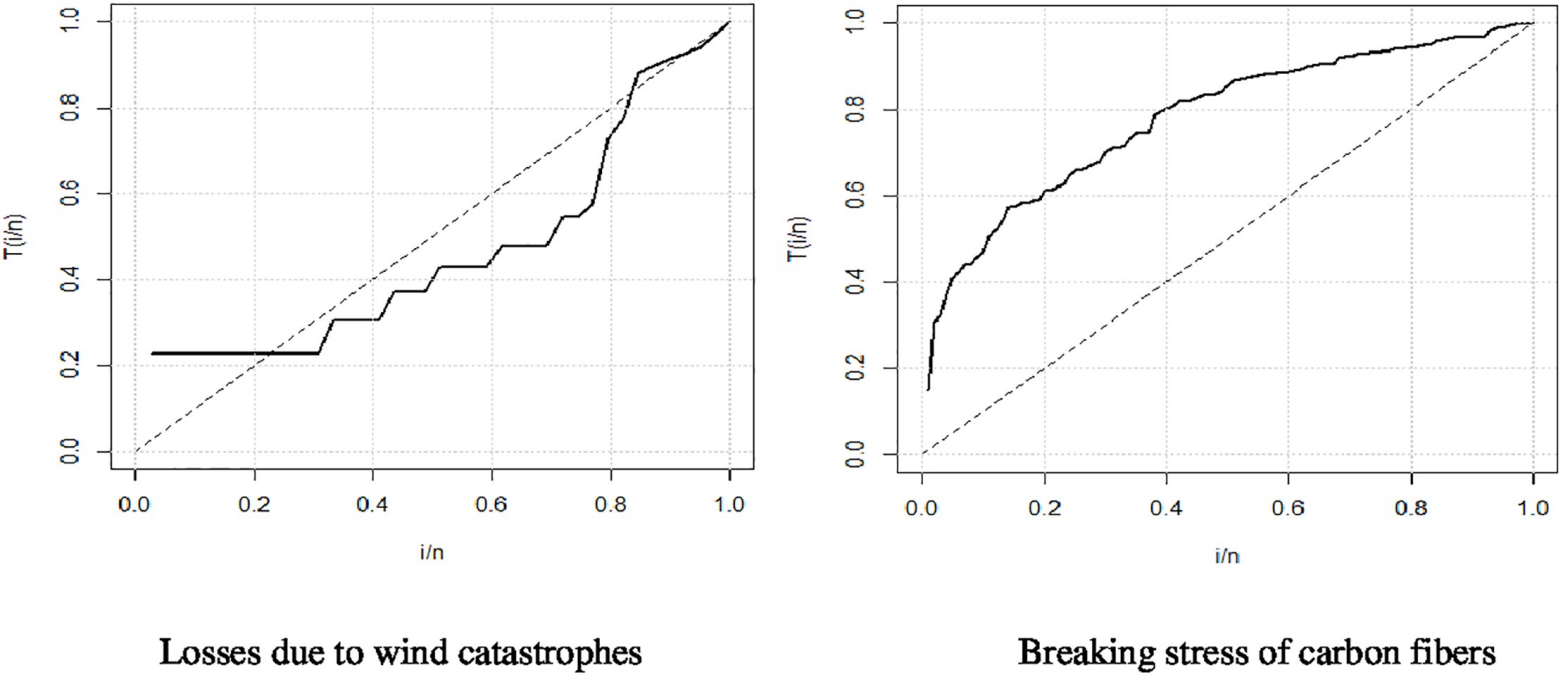
TTT plot for wind catastrophes and carbon fibers.

The TTT plots for the data (losses due to wind catastrophes and breaking stress of carbon fibers) are given in Fig 7. The graph clearly shows that the proposed distribution plays an important role both in monotonic and non-monotonic hazard rate shapes.

## Conclusion

In this paper, we presented a new modification of the Lomax distribution consisting of two parameters called Lomax exponential Distribution (LE). The statistical properties of the LE distribution are obtained including moments, entropy measures, hazard function, Survival function, median, mode, order statistics, etc. Furthermore, the parameters of the model are estimated using the maximum likelihood estimation method. Asymptotic confidence intervals of the parameters, based on MLE, have been constructed. In future, a study may be conducted to estimate the parameter of the proposed model using Bayesian approach. The behavior of the hazard rate function has been investigated. It is concluded that the Lomax exponential distribution can model data sets having both monotonically and non-monotonically hazard rate shapes. The paper also presents an application of the LE distribution by using two real data sets. The results based on the real-life data sets reveal that the proposed distribution is more flexible for the lifetime data sets and provide a better fit to the data sets as compared to other competing probability models including the Lomax distribution, Weibull Lomax distribution, and exponential Lomax distribution.

## Supporting information

S1 Data Losses due to wind catastrophes. [38] 2,2,2,2,2,2,2,2,2,2,2,2,3,3,3,3,4,4,4,5,5,5,5,6,6,6,6,8,8,9,15,17,22,23,24,25,27,32,43.
(DOCX)

S2 Data Breaking stress of carbon fibers. [18] 3.70,2.74,2.73,2.50,3.60,3.11,3.27,2.87,1.47,3.11,4.42,2.41,3.19,3.22,1.69,3.28,3.09,1.87,3.15,4.90,3.75,2.43,2.95,2.97,3.39,2.96,2.53,2.67,2.93,3.22,3.39,2.81,4.20,3.33,2.55,3.31,3.31,2.85,2.56,3.56,3.15,2.35,2.55,2.59,2.38,2.81,2.77,2.17,2.83,1.92,1.41,3.68,2.97,1.36,0.98,2.76,4.91,3.68,1.84,1.59,3.19,1.57,0.81,5.56,1.73,1.59,2.00,1.22,1.12,1.71,2.17,1.17,5.08,2.48,1.18,3.51,2.17,1.69,1.25,4.38,1.84,0.39,3.68,2.48,0.85,1.61,2.79,4.70,2.03,1.80,1.57,1.08,2.03,1.61,2.12,1.89,2.88,2.82,2.05,3.65.
(DOCX)

## References

[1] Ghitany M. E., Al-Awadhi F. A., Alkhalfan L. A. Marshall–Olkin extended Lomax- distribution and its application to censored data, Communications in Statistics, Theory and Methods, 2007; 36:1855–1866.

[2] Cordeiro G. M., Ortega E. M., Popovic B. V. The gamma-Lomax distribution, Journal of Statistical computation and Simulation, 2015; 85:305–319.

[3] Zografos. Konstantinos., Narayanaswamy B. On families of beta-and generalized gamma-generated distributions and associated inference. Statistical Methodology, 2009; 6(4): 344–362.

[4] Lemonte Artur J., Cordeiro Gauss M., Edwin M.M.O. On the additive Weibull distribution." Communications in Statistics-Theory and Methods, 2014; 43: 2066–2080.

[5] Lai C. D., Min Xie., Murthy D. N. P. A modified Weibull distribution. IEEE Transactions on reliability, 2003; 52 (1): 33–37.

[6] Lemonte Artur J., Gauss M. C. An extended Lomax distribution. Statistics, 2013; 47(4): 800–816.

[7] Ibrahim A.B., Moniem A., Hameed A. Exponentiated Lomax distribution International, Journal of Mathematical Education. 2012; 33(5):1–7.

[8] Ashour S. K., Eltehiwy M. A. Transmuted exponentiated Lomax distribution. Australian Journal of Basic and Applied Sciences, 2013; 7(7): 658–667.

[9] Merovci F., Puka L. Transmuted Pareto distribution. In Prob Stat Forum, 2014; 7:1–11.

[10] Khan Muhammad, S., Robert K., Hudson I. Characterizations of the transmuted inverse Weibull distribution. Anziam Journal. 2013; 55: 197–217.

[11] Tahir Muhammad H., et al The Weibull-Lomax distribution properties and applications. Hacettepe Journal of Mathematics and Statistics. 2015; 44(2): 461–480.

[12] Harris C. M. The Pareto distribution as a queue service discipline. Operations Research, 1968; 16(2):307–313.

[13] Atkinson A.B. and Harrison A.J. Distribution of Personal Wealth in Britain (Cambridge University Press, Cambridge, 1978).

[14] Hollanh O., Golaup A. and Aghvami A.H. Traffic characteristics of aggregated module downloads for mobile terminal reconfiguration, IEE proceedings on Communications, 2006; 135:683–690.

[15] Hassan A.S. and Al-Ghamdi A.S. Optimum step stress accelerated life testing for Lomax distribution, Journal of Applied Sciences Research, 2009; 5:2153–2164.

[16] Bryson M. C. Heavy-tailed distributions: properties and tests. Technometrics, 1974; 16(1):61–68.

[17] Ahsanullah M. Record values of Lomax distribution, Statistica Nederlandica, 1991; 41(1): 21–29.

[18] El-Bassiouny A. H., Abdo N. F., Shahen H. S. Exponential lomax distribution. International Journal of Computer Applications, 2015; 121(13).

[19] Afify A. Z., Nofal Z. M., Yousof H. M., El Gebaly Y. and M.,Butt N. S. The transmuted Weibull Lomax distribution: properties and application, Pakistan Journal of Statistics and Operation Research, 2015; 11: 135–152.

[20] Cordeiro G. M., Alizadeh M., Ramires T. G., and Ortega E. M. The generalized odd half-Cauchy family of distributions properties and applications, Communications in Statistics-Theory and Methods, 2017; 46: 5685–5705.

[21] Abd-Elfattah A.M., Alaboud F.M. and Alharby A.H. On sample size estimation for Lomax distribution, Australian Journal of Basic and Applied Sciences, 2007; 1: 373–378.

[22] Lemonte A. J., and Cordeiro G. M. An extended Lomax distribution, Statistics, 2013; 47: 800–816.

[23] Ahsanullah M. Record values of Lomax distribution, Statistica Nederlandica, 1991; 41(1): 21–29.

[24] Abd-Elfattah A.M., Alaboud F.M. and Alharby A.H. On sample size estimation for Lomax distribution, Australian Journal of Basic and Applied Sciences, 2007; 1:373–378.

[25] Al-Zahrani B., and Sagor H. The poisson-lomax distribution. Revista Colombiana de Estadística, 2014; 37: 225–245.

[26] Korkmaz M. c., and Genç A. I. A new generalized two-sided class of distributions with an emphasis on two-sided generalized normal distribution, Communications in Statistics-Simulation and Computation, 2017; 46: 1441–1460.

[27] Nasir M. A., Aljarrah M., Jamal F., and Tahir M. H. A new generalized Burr family of distributions based on quantile function. Journal of Statistics Applications and Probability, 2017; 6:1–14.

[28] Otunuga O. E. The Pareto-g Extended Weibull Distribution, 2017.

[29] Dias C. R., Alizadeh M., and Cordeiro G. M. The beta Nadarajah-Haghighi distribution. Hacettepe University Bulletin of Natural Sciences and Engineering Series B: Mathematics and Statistics, 2016.

[30] El-Bassiouny A. H., Abdo N. F., and Shahen H. S. Exponential lomax distribution, International Journal of Computer Applications, 2015; 121:(13).

[31] Ashour S. K., Eltehiwy M. A. Transmuted exponentiated Lomax distribution, Australian Journal of Basic and Applied Sciences, 2013; 7: 658–667.

[32] Chen Feng, Chen Suren, and Ma Xiaoxiang. Analysis of hourly crash likelihood using unbalanced panel data mixed logit model and real-time driving environmental big data. Journal of safety research, 2018; 65: 153–159. 10.1016/j.jsr.2018.02.010 29776524

[33] Chen Feng, and Chen Suren. Injury severities of truck drivers in single-and multi-vehicle accidents on rural highways. Accident Analysis & Prevention,2011; 43(5)): 1677–1688. 21658494 10.1016/j.aap.2011.03.026

[34] Chen Feng, Song Mingtao, and Ma Xiaoxiang. Investigation on the injury severity of drivers in rear-end collisions between cars using a random parameters bivariate ordered probit model. International journal of environmental research and public health; 2019; 16(14): 2632. 10.3390/ijerph16142632 PMC6678079 31340600

[35] Dong Bowen, et al "Investigating the Differences of Single-Vehicle and Multivehicle Accident Probability Using Mixed Logit Model. Journal of Advanced Transportation, 2018 10.1007/s11116-016-9747-x

[36] Chen Feng, Chen Suren, and Ma Xiaoxiang. Crash frequency modeling using real-time environmental and traffic data and unbalanced panel data models. International journal of environmental research and public health, 2016; 13(6): 609. 10.3390/ijerph13060609 PMC4924066 27322306

[37] Lomax K. Business failures: another example of the analysis of failure data. J Am Stat Assoc. 1987; 49: 847–852.

[38] Hogg R. and Klugman S.A. Loss Distributions. New York: Wiley; 1984.

