# Supplementary material for: Lomax exponential distribution with an application to real-life data
Source: PLoS One. 2019 Dec 11;14(12):e0225827. doi: 10.1371/journal.pone.0225827 (PMC6905582; doi:10.1371/journal.pone.0225827)
Supplement: S1 Data — [38] 2,2,2,2,2,2,2,2,2,2,2,2,3,3,3,3,4,4,4,5,5,5,5,6,6,6,6,8,8,9,15,17,22,23,24,25,27,32,43. (DOCX) [file pone.0225827.s001.docx]

Data set 1. Losses due to wind catastrophes

2,2,2,2,2,2,2,2,2,2,2,2,3,3,3,3,4,4,4,5,5,5,5,6,6,6,6,8,8,9,15,17,22,23,24,25,27,32,43.

[38] Hogg R. and Klugman S.A. Loss Distributions. New York: Wiley; 1984.
