# Supplementary material for: Lomax exponential distribution with an application to real-life data
Source: PLoS One. 2019 Dec 11;14(12):e0225827. doi: 10.1371/journal.pone.0225827 (PMC6905582; doi:10.1371/journal.pone.0225827)
Supplement: S2 Data — [18] 3.70,2.74,2.73,2.50,3.60,3.11,3.27,2.87,1.47,3.11,4.42,2.41,3.19,3.22,1.69,3.28,3.09,1.87,3.15,4.90,3.75,2.43,2.95,2.97,3.39,2.96,2.53,2.67,2.93,3.22,3.39,2.81,4.20,3.33,2.55,3.31,3.31,2.85,2.56,3.56,3.15,2.35,2.55,2.59,2.38,2.81,2.77,2.17,2.83,1.92,1.41,3.68,2.97,1.36,0.98,2.76,4.91,3.68,1.84,1.59,3.19,1.57,0.81,5.56,1.73,1.59,2.00,1.22,1.12,1.71,2.17,1.17,5.08,2.48,1.18,3.51,2.17,1.69,1.25,4.38,1.84,0.39,3.68,2.48,0.85,1.61,2.79,4.70,2.03,1.80,1.57,1.08,2.03,1.61,2.12,1.89,2.88,2.82,2.05,3.65. (DOCX) [file pone.0225827.s002.docx]

Data set 2. Breaking stress of carbon fibers.

3.70,2.74,2.73,2.50,3.60,3.11,3.27,2.87,1.47,3.11,4.42,2.41,3.19,3.22,1.69,3.28,3.09,1.87,3.15,4.90,3.75,2.43,2.95,2.97,3.39,2.96,2.53,2.67,2.93,3.22,3.39,2.81,4.20,3.33,2.55,3.31,3.31,2.85,2.56,3.56,3.15,2.35,2.55,2.59,2.38,2.81,2.77,2.17,2.83,1.92,1.41,3.68,2.97,1.36,0.98,2.76,4.91,3.68,1.84,1.59,3.19,1.57,0.81,5.56,1.73,1.59,2.00,1.22,1.12,1.71,2.17,1.17,5.08,2.48,1.18,3.51,2.17,1.69,1.25,4.38,1.84,0.39,3.68,2.48,0.85,1.61,2.79,4.70,2.03,1.80,1.57,1.08,2.03,1.61,2.12,1.89,2.88,2.82,2.05,3.65.

[18] El-Bassiouny, A. H., Abdo, N. F., Shahen H. S. Exponential lomax distribution. International Journal of Computer Applications, 2015; 121(13).
